# Comparative assessment of left atrial volume in healthy cats by two-dimensional and three-dimensional echocardiography

**DOI:** 10.1186/s12917-020-02473-6

**Published:** 2020-07-29

**Authors:** Janina Rauch, Michael Fehr, Martin Beyerbach, Stephan O. Hungerbuehler

**Affiliations:** 1 Small Animal Clinic, University of Veterinary Medicine Hannover, Foundation, Bünteweg 9, D-30559 Hannover, Germany; 2 Department of Biometry, Epidemiology and Information Processing, University of Veterinary Medicine Hannover, Foundation, Bünteweg 2, D-30559 Hannover, Germany; 3 Tiergesundheitszentrum Hungerbühler, Tierärztliche Klinik für Kleintiere Salzgitter, Gerichtsweg 3, 38229 Salzgitter, Germany

**Keywords:** Cat, Left atrium, Volumetry, RT3DE, RTTPE, LA, 2D, 3D

## Abstract

**Background:**

The left atrium (LA) is an important prognostic parameter in cardiac pathologies of cats. Its size is currently measured in one-dimensional methods, while human medicine considers two- and three-dimensional echocardiography as standard. The objectives of this study were to compare monoplane, biplane, triplane and real-time three dimensional echocardiography for volumetric measurement of the left atrium in healthy cats and establish a reference interval for further studies on cats with heart disease. Additionally, the influence of age, sex and weight on left atrial volume (LAV) was tested.

**Results:**

One dimensional monoplane Simpson method of discs (SMOD) in the right parasternal four chamber view (r4) and the left apical 2 chamber view (l2) as well as biplane SMOD had no significant difference for left atrial maximum volume (LAMax). They can be used as equivalent in future studies and one common reference range was set up (1.96 ± 0.54 ml). Those three methods produced significantly higher volumes than triplane echocardiography (RTTPE) and real time three dimensional echocardiography (RT3DE) using TomTec® software. LA volumetry with RTTPE and RT3DE-TomTec™ was more feasible than expected, but low RT3DE image quality was the main reason for excluding patients. Neither age nor weight had an influence on LA volume in healthy cats. Male LAV results were only slightly, but in 2D and RTTPE significantly higher than those of female cats with a range of + 10.46% to + 19.58%.

**Conclusions:**

Monoplane, biplane, triplane and real-time three dimensional echocardiography were feasible for LA volumetry in healthy cats and showed acceptable intra- and interobserver variability. One common LAMax reference range for monoplane r4, l2 and biplane SMOD was set up. Raw data can be used for LA volumes and does not need to be correlated with the cat’s weight or age. Male cats have only slightly but significantly larger atria than females in 2D and RTTPE. Therefore, under reservation, also sex related limit values were defined.

## Background

The left atrium (LA) is an important prognostic parameter in cardiac pathologies of cats [1–3]. LA size significantly increases with progressing severity of left sided cardiomyopathies [1, 3–7]. Furthermore, an expansion of LA leads to decelerated blood flow in the LA and in the left atrial appendage (LAA). This causes an increased risk for life threatening aortic thromboembolism in cats with dilatation of the LA [8, 9]. Left sided congestive heart failure that is detected too late can also lead to secondary pulmonary hypertension in cats [10].

Radiography and ECG are specific, but insensitive methods for evaluation of LA enlargement [11]. CMRI is golden standard for the measurement of LA in human medicine because it captures the most realistic asymmetrical structure, but the requirement of general anesthesia and high costs make MRI an unsuitable standard examination method in cats [12]. No comparable studies have been performed. The best non-invasive way to determine LA size and function in awake cats is via echocardiography [13]. Currently, one-dimensional (1D) diameter measurements are most commonly used in cats because they are simple and fast to perform [13]. Furthermore, the ratio between the LA diameter and the diameter of the aortic valve (LA/AO) is used to detect atrial dilatation [1, 13–21]. Recently, some authors have reported that pulmonary vein to pulmonary artery ratio variables were better factors than LA/AO for identifying cats with congestive heart failure [22].

The common disadvantage of these methods is the evaluation of a three-dimensional (3D) structure based on a single diameter. Consequently, there is a risk that a dilatation, which can occur in all 3 dimensions, will not be detected.

The quantification of the LA area and ratios of LA area and circumference measurements with the aorta are not commonly used, because mono- and multiplane volume measurements have shown the potential to be more reliable for early diagnosis of LA enlargement in cats, dogs and humans [5, 9, 14, 23–26]. The two most common techniques are area-length (A/L) and Simpson Method of Discs (SMOD). Each method can be used to calculate monoplane volume or biplane volume.

Monoplane SMOD measurement in the r4 and l4 plane was used for evaluation of LA size in two studies before and was proven to be a promising echocardiographic method for LA volumetry [5, 26].

The American Society of Echocardiography and the European Association of Cardiovascular Imaging recommend biplane SMOD over A/L, because it is based on less geometric assumptions [27]. There are no studies comparing A/L and SMOD or monoplane and biplane methods for LA volumetry in cats.

Triplane volumetric examination (RTTPE) is a three-dimensional method using 3 planes in a 60° angle at the same time. In left ventricle volumetry (LVV), RTTPE was proven to be more accurate and faster than SMOD when compared to gold standard cMRI in humans and dogs [28, 29]. There are no studies for evaluation of LA using RTTPE in veterinary medicine.

Real time three dimensional echocardiography (RT3DE) is the most modern ultrasound technique and generates a dynamic three dimensional volume block of the heart based on endocardial border detection. In human medicine, RT3DE using the software TomTec® provides results that underestimate cMRI calculations less than 2DE, as they capture asymmetrical LA expansion in contrast to 2DE [23, 30, 31]. Until now, there is very little experience with three dimensional LA volumetry in veterinary medicine. All available studies have been performed in dogs [32, 33]. LA volume measured by RT3DE differed significantly from LA Area, Diameter, LA/AO and M-Mode even after indexation to consider the influence of different body weights.

In summary, there is a lack of studies using volumetric examinations in feline cardiology. Since these methods are clearly superior to diameter measurements in humans and dogs, they are of high interest for feline medicine as well. The aim of this study was to evaluate 2DE, RTTPE and RT3DE for volumetric measurement of the left atrium in healthy cats and establish a reference interval for further studies on cats with heart disease. Additionally, the influence of age, sex and weight on left atrial volume (LAV) was tested.

## Results

### Image acquisition

Out of 72 cats, 50 animals approved to be suitable for data acquisition. Insufficient 3DE loop quality was the main reason for dismissing patients. In these 20 cats, the resolution of the 1DE and 2DE frames was adequate, but the 3DE data was too blurred to be evaluated. In two patients, the ECG was incomplete and therefore prohibited correct analysis. The results are summarised as mean value ± standard deviation in Table 1*.* Significant differences between the measurement techniques are shown in Table 2.

**Table 1:**
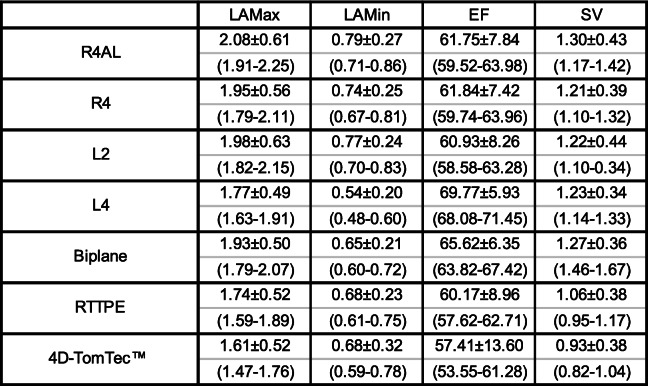
Overview of left atrial volumes (LAV Excluding Appendage): left atrial volumes in healthy cats (*n* = 50) measured with different methods. Data is presented as mean ± standard deviation in millilitre (ml) and as 95% prediction interval in the brackets (ml)

Abbreviations: *Max* Left atrial maximum volume, *Min* Left atrial minimal volume, *EF* Ejection Fraction, *SV* Stroke Volume, *R4AL* right parasternal 4 chamber view measured with 2D Area/Length method, *R4* 2D monoplane Simpson method of discs, *L2* left apical two chamber view, *L4* left apical four chamber view, *Biplane* L2 and L4 combined to biplane volumetry, *RTTPE* Real Time Triplane Echocardiography, 4D-TomTec™ analysing software

**Table 2:** Overview of *P*-Values for all left atrial (LA) measurement methods

Methods	LAMax	LAMin	EF	SV
R4 vs R4AL	< 0.0001	< 0.0001	**0.5814**	< 0.0001
R4 vs L2	**0.6690**	**0.3364**	**0.5530**	**0.9399**
R4 vs L4	0.0126	< 0.0001	< 0.0001	**0.7380**
R4 vs Biplane	**0.7009**	0.0045	0.0026	**0.3092**
R4 vs RTTPE	0.0042	**0.0914**	**0.3076**	0.0112
R4 vs 4DTomTec™	0.0001	**0.2473**	0.0315	< 0.0001
L2 vs L4	0.0021	< 0.0001	< 0.0001	**0.3003**
L2 vs Biplane	**0.0883**	< 0.0001	< 0.0001	**0.0541**
L2 vs RTTPE	< 0.0001	0.0006	**0.5378**	0.0005
L2 vs 4DTomTec™	< 0.0001	**0.0869**	**0.1030**	< 0.0001
L4 vs Biplane	< 0.0001	< 0.0001	< 0.0001	**0.1972**
L4 vs RTTPE	**0.5752**	< 0.0001	< 0.0001	0.0004
L4 vs 4DTomTec™	0.0185	0.0007	< 0.0001	< 0.0001
Biplane vs RTTPE	< 0.0001	**0.3309**	< 0.0001	< 0.0001
Biplane vs 4DTomTec™	< 0.0001	**0.5469**	< 0.0001	< 0.0001
RTTPE vs 4DTomTec™	0.0135	**0.9260**	**0.1949**	0.0044

Comparison of left atrial maximum and minimum volumes (LAMax/LAMin), ejection fraction (EF) and stroke volume (SV) in 50 healthy cats measured with 7 echocardiographic methods: right parasternal 4 chamber view measured with 2D Area/Length method (R4AL) and 2D monoplane Simpson method of discs (R4), left apical two chamber view (L2) and four chamber view (L4) measured with 2D monoplane Simpson method of discs, L2 and L4 combined to biplane volumetry (Biplane), Real Time Triplane Echocardiography (RTTPE), 4D-TomTec™ analysing software. Mean differences between the measurement-techniques were evaluated with a t-Test. *P* > 0.05 was evaluated as significant similarity and marked as bold letters

### Influence of age, sex and weight

The influence of age and weight on the LAV was calculated with the coefficient of determination R^2^. The results for age (range of 0.004–0.25) as well as for weight (range of 0.002–0.17) were below 0.26 with all methods. For LAMax, the influence of weight had even lower R^2^ values than that of age. The results for weight did not exceed 0.17 (R4AL 0.0350, R4 0.0352, L2 0.1206, L4 0.1509, Biplane 0.1661, RTTPE 0.0816, 4D-TomTec™ 0.0348). Also with the influence of age, no clear pattern could be distinguished between the methods (R4AL 0.0358, R4 0.0379, L2 0.1534, L4 0.2125, Biplane 0.2134, RTTPE 0.1793, 4D-TomTec™ 0.1473). The results are summarized in Tables 3 and 4.

**Table 3:** Coefficient of determination (R2) to evaluate the influence of age on left atrial volumes

Method	LAMax	LAMin	EF	SV
R4AL	0.0358	0.0227	0.0138	0.0297
R4	0.0379	0.0247	0.0091	0.0297
L2	0.1534	0.1589	0.0105	0.0876
L4	0.2125	0.1174	0.0048	0.2097
Biplane	0.2134	0.1627	0.0022	0.1664
RTTPE	0.1793	0.1044	0.0101	0.1580
4D-TomTec™	0.1473	0.0361	0.0186	0.1242

Coefficient of determination (R2) to evaluate the influence of age on left atrial (LA) maximum (LAMax) and minimum (LAMin) volume as well as on the ejection fraction (EF) and stroke volume (SV) in 50 healthy cats measured with 7 echocardiographic methods: right parasternal 4 chamber view measured with 2D Area/Length method (R4AL) and 2D monoplane Simpson method of discs (R4), left apical two chamber view (L2) and four chamber view (L4) measured with 2D monoplane Simpson method of discs, L2 and L4 combined to biplane volumetry (Biplane), Real Time Triplane Echocardiography (RTTPE) and 4D-TomTec™ analysing software

**Table 4:** Coefficient of determination (R2) to evaluate the influence of weight on left atrial volumes

Method	LAMax	LAMin	EF	SV
R4AL	0.0350	0.1289	0.0903	0.0010
R4	0.0352	0.1359	0.1123	0.0007
L2	0.1206	0.1321	0.0126	0.0658
L4	0.1509	0.1275	0.0007	0.1196
Biplane	0.1661	0.1402	0.0044	0.1202
RTTPE	0.0816	0.0590	0.0024	0.0642
4D-TomTec™	0.0348	0.0080	0.0120	0.0307

Coefficient of determination (R2) to evaluate the influence of weight on left atrial (LA) maximum (LAMax) and minimum (LAMin) volume as well as on the ejection fraction (EF) and stroke volume (SV) in 50 healthy cats measured with 7 echocardiographic methods: right parasternal 4 chamber view measured with 2D Area/Length method (R4AL) and 2D monoplane Simpson method of discs (R4), left apical two chamber view (L2) and four chamber view (L4) measured with 2D monoplane Simpson method of discs, L2 and L4 combined to biplane volumetry (Biplane), Real Time Triplane Echocardiography (RTTPE) and 4D-TomTec™ analysing software

Male LAV results were consistently slightly higher than those of female cats with a range of + 10.46% to + 19.58% between the different methods. The overall mean difference of LAV measurements between males and females for LAMax was 0.30 ml with a standard deviation of 0.73 ml, and for LAMin it was 0.14 ml with a standard deviation of 0.40 ml. The differences were significant in monoplane and biplane methods, but not using three dimensional echocardiography. The results are summarized in Table 5.

**Table 5:** Comparison of left atrial volumes in healthy male (*n* = 26) and female (*n* = 24) cats

	LAMax				LAMin			
	Male	Female	MD ml	MD %	Male	Female	MD ml	MD %
R4	2.26	1.89	**0.37**	**19.58**	0.88	0.68	**0.19**	**27.79**
L2	2.11	1.78	**0.33**	**18.54**	0.83	0.64	**0.18**	**28.13**
L4	2.14	1.81	**0.33**	**18.01**	0.83	0.70	**0.13**	**18.76**
Biplane	1.90	1.63	**0.28**	**17.11**	0.59	0.48	**0.12**	**24.23**
RTTPE	2.09	1.76	**0.33**	**18.75**	0.72	0.59	0.13	22.03
4D-TomTec™	1.84	1.63	0.21	12.88	0.73	0.62	0.11	17.74

Comparison of left atrial maximum and minimum volumes (LAMax/LAMin) in male (*n* = 26) and female (*n* = 24) healthy cats measured with 7 echocardiographic methods: right parasternal 4 chamber view measured with 2D Area/Length method (R4AL) and 2D monoplane Simpson method of discs (R4), left apical two chamber view (L2) and four chamber view (L4) measured with 2D monoplane Simpson method of discs, L2 and L4 combined to biplane volumetry (Biplane), Real Time Triplane Echocardiography (RTTPE) and 4D-TomTec™ analysing software. Mean difference (MD) of LAV between male and female was evaluated with a t-Test. Significant differences (*p* < 0.05) are marked as bold letters

### Comparison of monoplane SMOD and Area/Length

R4 was measured with the Simpson as well as the A/L method. They proved to be significantly different (*p* < 0.05). As a consequence, the two methods cannot be used equivalently. The American Association of Echocardiography recommends SMOD over A/L for LA volumetry in human medicine [12]. Therefore, the Simpson technique was used to calculate the volumes in the other monoplane and biplane views in this study.

### LAMax and LAMin

#### Monoplane and biplane measurements of LAMax

R4 had no significant difference to l2 (*p* = 0.6690, bias = 0.032) and biplane SMOD (*p* = 0.7009, bias = − 0.025). Those three methods resulted in significantly higher volumes than RTTPE and 4D-TomTec. L4 showing the lowest monoplane volumes differed from all 2D methods, but not from RTTPE (*p* = 0.5752, bias = − 0.0306).

#### 3D measurements of LAMax

RTTPE and 4D-TomTec™ differed significantly (*p* = 0.0135, bias = − 0.1266). 4D-TomTec™ quantified the lowest LAMax volumes of all methods. RTTPE results were significant lower than r4 (*p* = 0.0042, bias = − 0:025), l2 (*p* = < 0.0001, bias = − 0.2442) and biplane volumes (*p* = < 0.0001, bias = − 0:1872), but had no significant difference to l4 (*p* = 0.5752, bias = − 0.0306).

#### Monoplane and biplane measurements of LA min

LAMin using r4 and l2 had no significant difference and (*p* = 0.3364) produced the highest results of all methods. L4 results were significantly lower than all other volumes. Biplane differed significantly from all monoplane measurements with higher results than l4 and lower results than l2 and r4. No significant difference was found between l2 and 4D-TomTec™ (*p* = 0.0869), as well as between R4 and RTTPE (*p* = 0.0914) and 4D-TomTec™ (*p* = 0.2473).

#### 3D measurements of LAMin

RTTPE and 4D-TomTec™ resulted in exactly the same mean value (*p* = 0.9260). Furthermore, there was no significant difference between 3D, biplane and l2 volumes. L4 resulted in significantly lower and r4 in significantly higher volumes than 3D.

#### EF

Again, r4 and l2 showed no significant difference (*p* = 0.5530). The highest EF was measured with l4, which differed significantly from all other methods. Biplane results were significantly different from all other methods as well. 3D measurements did not differ significantly from r4 and l2.

#### SV

SV values of r4, l2, l4 as well as biplane showed no significant differences with nearly identical mean raw values. RTTPE resulted in significantly lower values than those methods. However, the significantly lowest mean SV was measured with 4D-TomTec™.

### Inter- and Intraobserver variability

Inter- and intraobserver variability for LAMax and LAMin was expressed as CV in percent and summarized in Table 6. Interobserver variability was generally higher in LAMin than in LAMax measurements. Biplane (5.59%) and RTTPE (5.55%) resulted in the lowest interobserver variability for LAMax, while R4 (20.28%) had by far the highest values. Interobserver variability was generally high for LAMin and LAMax, ranging between 25.91% and 47.06%.

**Table 6:** Inter- and intraobserver variability

	CV interobserver (%)		CV intraobserver (%)	
	LAMax	LAMin	LAMax	LAMin
Excluding Appendage				
R4	20.28	27.19	28.72	33.78
L2	13.30	30.87	31.82	31.17
L4	9.52	16.81	27.68	37.04
Biplane	5.59	18.22	25.91	32.31
RTTPE	5.55	15.25	29.89	33.82
4D-TomTec™	16.63	28.93	32.30	47.06

Inter- and intraobserver variability for left atrial maximum (LAMax) and LA minimum (LAMin) expressed as coefficient of variation (CV) in percent (%). LAMax and LAMin were measured with 6 echocardiographic methods: 2D monoplane Simpson method of discs (R4), left apical two chamber view (L2) and four chamber view (L4) measured with 2D monoplane Simpson method of discs, L2 and L4 combined to biplane volumetry (Biplane), Real Time Triplane Echocardiography (RTTPE) and 4D-TomTec™ analysing software

### Analysis time

The mean amount of time necessary for completing the various techniques of offline volumetry are summarized in Table 7. Basically, the time rises with the number of dimensions measured. The only exception is the biplane method, which took 10 s longer than the RTTPE technique. 4D-TomTec™ took 8.4 times as long as the simple monoplane diameter measurement and 4.1 times as long as the monoplane Simpson method. R4AL and R4 values were simultaneously calculated because they are based on the same measurements. Therefore their durations are identical. Apart from these two, there was a significant difference between measurement durations of each method (*p* = < 0.005).

**Table 7:** Comparison of measurement time in seconds

Analysis Time	Mean	SD	Min	Max
R4AL	20.62	2.18	17.09	24.92
R4	20.62	2.18	17.09	24.92
L2	22.03	1.85	18.36	28.47
L4	23.50	2.83	18.27	29.53
Biplane	49.67	4.04	41.72	55.76
RTTPE	39.66	1.64	37.21	43.11
4D-TomTec™	85.29	9.80	76.62	106.48

Mean, standard deviation (SD), minimum (Min) and maximum (Max) duration in seconds (sec.) for left atrial volumetry in 50 healthy cats measured with 7 echocardiographic methods: right parasternal 4 chamber view measured with 2D Area/Length method (R4AL) and 2D monoplane Simpson method of discs (R4), left apical two chamber view (L2) and four chamber view (L4) measured with 2D monoplane Simpson method of discs, L2 and L4 combined to biplane volumetry (Biplane), Real Time Triplane Echocardiography (RTTPE) and 4D-TomTec™ analysing software. There was a significant difference between measurement durations of each method (*p* = < 0.005)

## Discussion

The main finding of this study is that it is possible to measure two, three and four dimensional volumes of the left atrium in healthy cats. Apart from that, reference values were established for all methods and one common LAMax reference value could be defined for l2, r4 and biplane SMOD. Bodyweight and age did not have a significant effect on the results, while male cats produced slightly larger values than females.

### Volumetry

Echocardiography in awake cats is not easy because of the animal’s movement and small LA size. Artifacts due to movement and therefore inadequate quality of 3DE data were the main reason for excluding patients. Out of 72 cats, 50 animals approved to be suitable for data acquisition. In 20 cats, the resolution of the 1DE and 2DE frames was adequate, but the 3DE data was too blurred to be evaluated. In two patients, the ECG was incomplete and therefore prohibited correct analysis. Furthermore, the small size of a cat’s atrium requires very high image resolution. The high heart rate makes a high frame rate necessary to ensure adequate temporal resolution.

#### Monoplane methods

Monoplane r4, l2 and biplane resulted in mean values that are equal to the first decimal place and did not show statistic difference for LAMax (*p* > 0.05). Therefore they can be used equivalently for LAMax evaluation in future studies. One reference range for all three methods was set up: 1.96 ± 0.54 ml (Table 1). Those three methods produced significantly higher volumes than RTTPE and 4D-TomTec™. This accords with former findings in human medicine, where monoplane 2D methods measured higher LAV than 3DE due to geometric assumptions instead of endocardial border detection [12, 34, 35]. R4 monoplane SMOD values in a comparative study were higher (2.24 cm^3^ mean value) than those in this study (1.95 cm^3^ mean value). However, only 21 cats were included and only 11 were European Short Hair [5]. L4 had lower results and therefore differed from all 2D methods except for RTTPE (*p* = 0.5752). The significant difference between l2 and l4 LA volumes is due to the ovoid LA shape and therefore different diameters. This can be seen well in 3D-images, where both planes are displayed concurrently (Fig. 2, Fig. 3). No veterinary study exists on the comparison of l2 and l4. We expected r4 and l4 to be similar, as LV-volumes in dogs using these planes were the same [29]. However, there was a small (0.2 ml) but significant (*p* = 0.126) difference. This could be caused by a slightly oblique angle and the ovoid shape of the LA. Because of the flattened position of the feline heart, an optimal long axis view is difficult to achieve in some cats. A similar effect is described in older humans caused by alterations of the position of the diaphragm [12]. Based on our results, two limit values are necessary for monoplane LA. This finding is shared in a comparable new study in cats, where the mean values for r4 (1.95 ml) and l4 (1.77 ml) LA SMOD are exactly the same as ours [26].

Also for LAMin, results from the l4 plane were significantly lower than all other monoplane volumes and R4 and l2 did not differ significantly (*p* = 0.3364). In a comparative study with higher monoplane SMOD LAMax values, the LAMin values are higher too (range of 0.73 to 1.22 ml). These consistently higher results justify the assumption that measurements were performed more generously than in this paper and inter-observer variability mismatches [5]. In a new comparable study with 162 cats, the mean monoplane SMOD measurements in the l4 plane equal ours to the first decimal space and in the r4 plane they only differ by 0.1 ml. It was suggested that LAMin was superior to LAMax to distinguish cardiomyopathy and congestive heart failure [26]. As LAMin results are very low (0.5 to 0.8 ml), the parameter appears to be prone-to-error. Therefore we do not concur and do not recommend LAMin over LAMax.

EF and SV have large standard deviations (SD) in all methods. This can be explained by the small volumes, where minor variation has major impact. In an already mentioned study on monoplane LA volumetry in cats, EF had similarly large SD. It generally was decreased in cats with cardiac pathologies in comparison to healthy ones, but could not distinct decompensated from asymptomatic HCM [5]. EF therefore seems unsuitable as sole indicator for LA enlargement in cats. In dogs it was also proven, that EF tends to vary widely between animals and measurement techniques and is discouraged as independent marker for the left atrium [32]. In humans, LA has greater volume (22–52 ml) and results are advised to be indexed to body surface area, which leaves EF and SV without large SD [12].

#### Biplane method

Since biplane SMOD did not show significant difference to LAMax results of monoplane r4 and l2, measuring two planes does not provide additional benefit in healthy cats. In human medicine, no significant difference between monoplane and biplane SMOD was found in healthy patients as well as patients with cardiac diseases [36]. Nevertheless, the American Association of Echocardiography and the European Association of Cardiovascular Imaging recommend biplane SMOD as standard technique for LA volumetry, because monoplane SMOD formula assumes circular LA shape in the short axis view, which is not always accurate [12]. The absent advantage of biplane over monoplane SMOD in healthy cats may be explained by the very small volumes. Mean values of l4 and l2 differ significantly, but by only 0.2 ml and are obviously relativized in the SMOD formula. Additionally, biplane SMOD requires significantly more time for retrograde measurement than monoplane measurement. In summary, monoplane SMOD appears to be the preferable alternative to biplane SMOD for 2D LA volumetry in healthy cats. .

#### Triplane echocardiography

LAMax results were significantly lower than those of monoplane (r4 and l2) and biplane SMOD. These findings are contrary to LV volumetry in dogs, where RTTPE values were significantly higher than monoplane SMOD and identical with CMRI [29]. However, LA and LV have a different shape, which makes results and relations not comparable. In cats, no comparison of RTTPE and cMRI has been done yet. Therefore, it also has to be considered, that maybe l2 and r4 are false high and RTTPE is not false low. This could be explained by the longitudinal-oval LA shape. The automatically generated discs are circular since the method was originally designed for LV volumetry. Circular discs with l2 diameter would project beyond the outer rim of the oval atrium in every layer and produce false high results. RTTPE consequently may capture one plane with high volume (l2) and two planes with significantly lower volume (l3, l4).

LAMin was difficult to measure especially with 3D methods, due to limited image quality and barely options for adaption during analysis, caused by the small size of the normal feline left atrium. The manual border detection was more difficult than at monoplane measurements due to the low frequency probe. RTTPE is inferior to 2DE, but superior to RT3DE in terms of spatial resolution. Out of the 20 cats that were excluded from measurements due to too low image quality, only 4 were dismissed because of problems with RTTPE loops. Frame rate was above 40 fps in both 3DE methods, but above 60 in 2DE. The necessity of a special probe has to be rated negatively in terms of costs and practicability caused by the great size and weight. An advantage of RTTPE is the fast retrograde measurement. It only took twice as long as monoplane and even 10 s less than biplane SMOD. From all these reasons, it can be assumed, that RTTPE works well for LAV in healthy cats and using three planes perhaps provides more realistic results than mono- or biplane SMOD, but further studies are required for verification by comparison with cMRI.

#### Real time three dimensional echocardiography

RT3DE is supposed to be the most realistic and best echocardiographic method to capture the true LAV. In human LA volumetry, RT3DE has excellent correlation with cMRI and usually larger volumes than 2DE [12, 37, 38]. In our study, 4D-TomTec™ quantified the lowest LAMax volumes of all methods. This is also the case in LA and LV volumetry in dogs [29, 33]. One possible explanation is the low image resolution due the low frequency probe. This decreased the quality of the automatic endocardial border detection and impeded the manual corrections, since the agitated endocardial border seemed broadened. This also led to the longest measurement-duration of all compared methods. An additional problem was the temporal resolution which could have caused deviating results too. 3DE achieved frame rates of approximately 40–50 fps, which equals 12 to 20 images per heartbeat at a cat’s heartrate of 120 to 240 per minute. In human medicine a frame rate of 30 is reached for a heartrate of 60 to 100 beats per minute [39]. The Vivid E9^1^ is able to create a volume block by using multiple subvolumes of ECG triggered consecutive R-R cycles, to attain higher frame rates and therefore better resolution. However, because of the small amplitude of cat’s ECGs, the software had problems recognising the R-peak and produced irregular R-R distances. A reliable fusion of subvolumes could therefore not be performed.

Maybe the evaluation of LA with 3DE is less problematic in cats with cardiac pathologies and dilated atria. This will have to be verified in further studies. Currently, 3DE is limited for LA evaluation in cats due to the tiny LA size, high heart rates, the demand for a special probe and analysis software, time consuming retrograde measurement and suboptimal image resolution.

### Influence of age, sex and weight

The coefficient of determination R^2^ was below 0.25 at all times for all methods when calculated for weight as well as for age. We concluded that LAV is not dependent on age and weight and raw data was used instead of correlated values. The finding concurs with data from human medicine. In a healthy human, LA size does not alter until the age of 80 [40]. The influence of age was never tested in cats before, but the effect of weight on LA has earlier been described as significant [15, 41–43]. However, these results were calculated on the base of M-Mode or LAD data and are therefore not comparable with our volumetry results.

In Human Medicine, it is recommend to index results to body surface area [12]. The influence of body surface area on LAV is also proven in dogs [44], but also allometric scaling to the bodyweight was justified [45–47].

In our study, the population almost exclusively consists of European Shorthair cats and the covered weight range is very narrow (4.42 ± 1.41 kg) which could explain the lack of influence over LAV. Maybe results would have been different if the tested population would have included more animals from large breeds (e.g. Maine Coons).

Male LAV results were slightly higher than those of female cats with a range of + 10.46% to + 19.58%. The differences were significant in monoplane and biplane methods. These results go well with former findings in cats and humans [12, 15, 41]. Nevertheless, a cat’s LAMax is 1.95 ± 0.56 ml with monoplane SMOD (r4), so a plus of 20% would only mean a difference of approximately 0.2 ml. Under reservation, gender specific reference values were established, but further studies in cats with LA Dilatation are necessary to evaluate, if gender specific limit values are really required.

### Inter- and Intraobserver variability

Generally, CV is remarkably high. This is probably due to the very low mean value and the high standard deviation in all methods. In a paper evaluating LA volumes in 40 dogs, CV was similarly high because of the same reason [48]. No clear distinction can be drawn between CV of either left apical and right parasternal views or the different measurement methods. Further studies in cats with cardiac pathologies are necessary to evaluate if inter- and intraobserver variability improves with pathologically enlarged LA volumes.

## Limitations

LAV was not measured with the golden standard cMRI. Therefore it is not possible to determine which echocardiographic method is the most realistic and reliable method. On the other hand it was never the aim of the study to elect the best method, but to evaluate which methods are possible, similar in their results and suitable for everyday use. Secondly, LA volumes in healthy cats are very small. There is a chance, that measurements are slightly more imprecise and more susceptible to minor variations than in LV measurements. Possibly, volumetry in cats with cardiac pathologies and dilated LA is easier and faster to perform. This of course requires confirmation by further studies. Also, we did not test the cats for hyperthyroidism. The disease primarily affects LV measurements and all cats were free of medical history, but nevertheless it would have rounded off the anamnesis if we had tested the blood value. Finally, the study is limited due to the small number of examined cats. To set up reference values by confidence intervals, it is advised to include at least 120 animals [49]. We included only 50 cats, but used mean ± SD, which is the recommended way to present truthful values from group of 40 to 120 participants [50].

## Conclusion

This study shows that monoplane, biplane, RTTPE and 4D-TomTec™ are applicable methods to assess left atrial volume in healthy cats. Most results differentiate significantly and therefore cannot be directly compared or exchanged in future studies. Only monoplane l2, r4 and biplane SMOD do not differ significantly and can be understood as equivalent. Furthermore, raw volume data can be used and does not need to be correlated with the cat’s weight or age. Male cats have significantly larger atria than females, therefore the set-up of sex related limit values for LA enlargement seemed legitimate.

## Methods

This study was approved by the Ethical Committee of the University of Veterinary Medicine Hannover and not classified as animal experiment as data was collected during regular cardiac examination.

### Animals

For this study, 50 unsedated cats underwent echocardiographic examination (26 male, 24 female, 6.48 ± 3.05 years, 48 European Shorthair, 1 Maine Coon and 1 Burmese, median weight of 4.42 ± 1.41 kg). The animals were patients of the Small Animal Hospital of the University of Veterinary Medicine Hannover. First, all 50 cats underwent clinical examination and blood pressure measurement. Then, the echocardiographic examination was performed. Following inclusion requirements had to be met: 1. The anamnesis was without history of cardiac pathologies, hypertonia, syncope and dyspnoea, 2. The clinical examination was clear of pathological findings, 3. The systolic blood pressure did not exceed 160 mmHg, 4. The echocardiography and ECG did not show any abnormalities, 5. The anteroposterior diameter of the left atrium, measured in the right parasternal long axis, was below 1.6 cm, and 6. The LA/AO ratio was below 1.4 in the right parasternal short axis view.

### Echocardiography

All echocardiographic exams were performed by one experienced veterinarian (S.O.H.) with a commercially available ultrasound unit^1^. It was equipped with a 12 S-D Phased Array probe for 1DE and 2DE ultrasonography (4–12 MHz) and a 3 V Matrix Array probe for 3D examination (2.5–3.6 MHz). The cats were laid in right lateral recumbency for the right parasternal long and short axis view and in left lateral position for left apical 2, 3 and 4 chamber view. The recommendations for cardiac chamber quantification by echocardiography of the American Society of Echocardiography were followed as closely as possible in the performance of the different techniques and are described in the chapters below [12]. Also, the ultrasound units’ ECG was attached and displayed during the examinations. For every plane, loops of at least three consecutive heart beats were recorded and saved for offline analysis. All data was transferred to a workstation^2^ for offline analysis.

#### Measurement of left atrial diameter and LA/AO

LAD measurement was performed in the same way the reference value of 1.6 cm was described [8, 11]. Both atria, both ventricles and the mitral and tricuspid valve were completely displayed. LAD was measured in ventricular end systole, defined as the moment exactly before mitral valve opening (LAMax) [9].

LA/AO measurement was performed in the right parasternal short axis view with the ‘Swedish method’ as described in dogs [51]. A limit value of 1.4 was used to classify healthy animals [1, 14]. The inner-edge to inner edge measurement of LA and Aortic valve was performed in early ventricular diastole, defined as the frame directly after closure of the aortic valve.

#### Left atrial volumetry

Left atrial maximum volume (LAMax) was measured directly before opening of the mitral valve, in ventricular end systole, one or two frames after the ECG’s T-wave. Left atrial minimum volume (LAMin) was measured directly after closing of the mitral valve at the end of LV diastole, one or two frames after the ECG’s P-wave. LAMax and LAMin, ejection fraction (EF) and stroke volume (SV) were measured with monoplane l2, l4 and r4, as well as biplane, RTTPE and 4D-TomTec™.

#### Monoplane examination

**Fig. 1:**
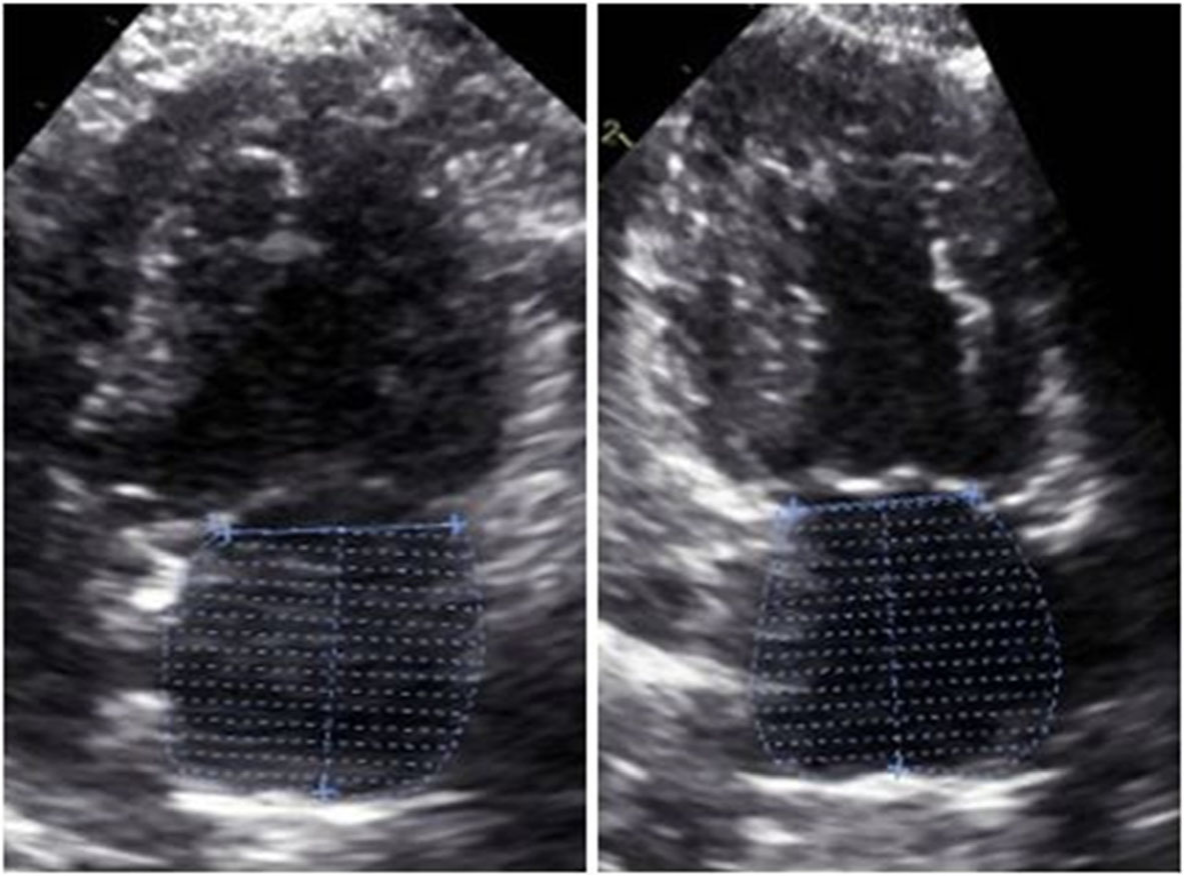
Left atrium (LA) volume measurement using monoplane Simpson method of discs (SMOD): Representative images of left atrial maximum volume (LAMax) measurement in a healthy cat with SMOD in left apical 4 chamber view (left) and left apical 2 chamber view (right)

For two-dimensional monoplane LA volumetry, the right parasternal 4 chamber view (r4), as well as the left apical 2 (l2) and 4 (l4) chamber views were displayed (Fig. 1) [12]. Attention was paid to avoid foreshortening by depicting the maximum (LV) length, the whole LA and in the l2 plane also the complete LAA. After LAMax and LAMin were assessed with Area/Length (A/L) for r4 and SMOD for r4, l4 and l2, the program automatically calculated EDV, ESV, ejection fraction (EF) and stroke volume (SV). SV describes the output during one systole in millilitres (ml). EF is the percentage of blood volume that is ejected by the left atrium during one heartbeat.

#### Biplane examination

For biplane examination the corresponding l2 and l4 planes were combined according to the recommendations of the American Society of Echocardiography [12].

#### Real time triplane echocardiography (RTTPE)

**Fig. 2:**
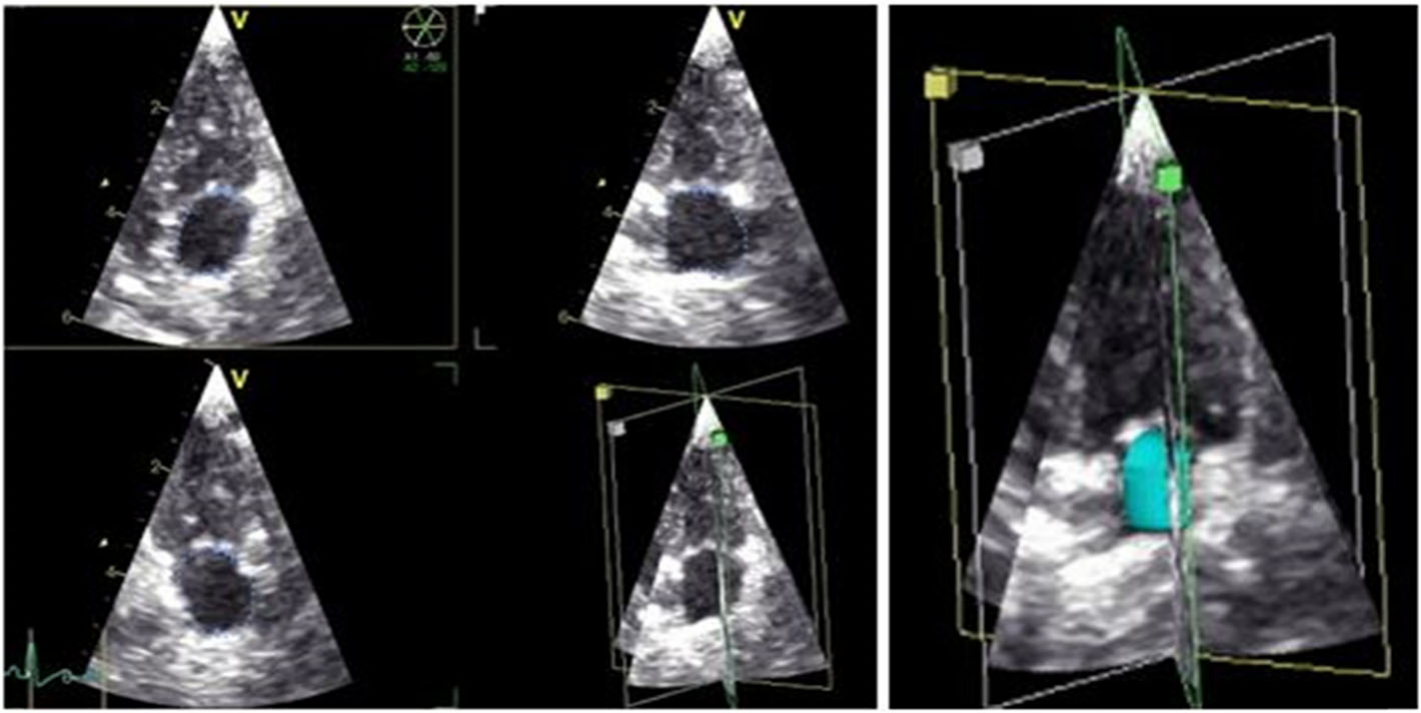
Left atrium (LA) volume measurement using Real Time Triplane Echocardiography (RTTPE): Representative images of left atrial maximum (LAMax) volumetry in a healthy cat with RTTPE (left). The atrium is concurrently displayed in all three angles. The analysis result is shown as three dimensional dynamic reconstruction of LA (right)

The RTTPE uses three planes, which are in an angle of 60° to each other. Based on the measurements in each of these planes, the software calculates a dynamic 3D reconstruction of the LA volume during the measured heart phase. This method was performed in the left apical axis of the heart. First, l4 view without signs of foreshortening was adjusted and then corrected until the concurrently displayed l2 and l3 planes depicted the entire LA including LAA. The width and depth of the frame was adjusted for closest capture of the left atrium to achieve frame rates above 40 frames per second (fps). The data sets were transferred to the workstation and measured later on. The definition of the time points for measurement were the same as described for the 2DE methods. At the beginning of offline analysis, the moment of LAMax was adjusted. Then, LA was traced along the endocardial border from the septal to the parietal mitral valve annulus in all three planes. The contour was automatically closed by connecting the opposite segments at mitral valve level. Attention was paid to exclude the pulmonary trunk as well as LAA. Next, the moment of LAMin was selected and the three planes were traced in the same pattern. Derived from these 6 measurements, the program automatically generated a dynamic reconstruction of the LA for the current heart cycle (Fig. 2). Additionally, LAMax, LAMin, EF and SV were calculated.

#### Real-time three dimensional echocardiography with border detection

**Fig. 3:**
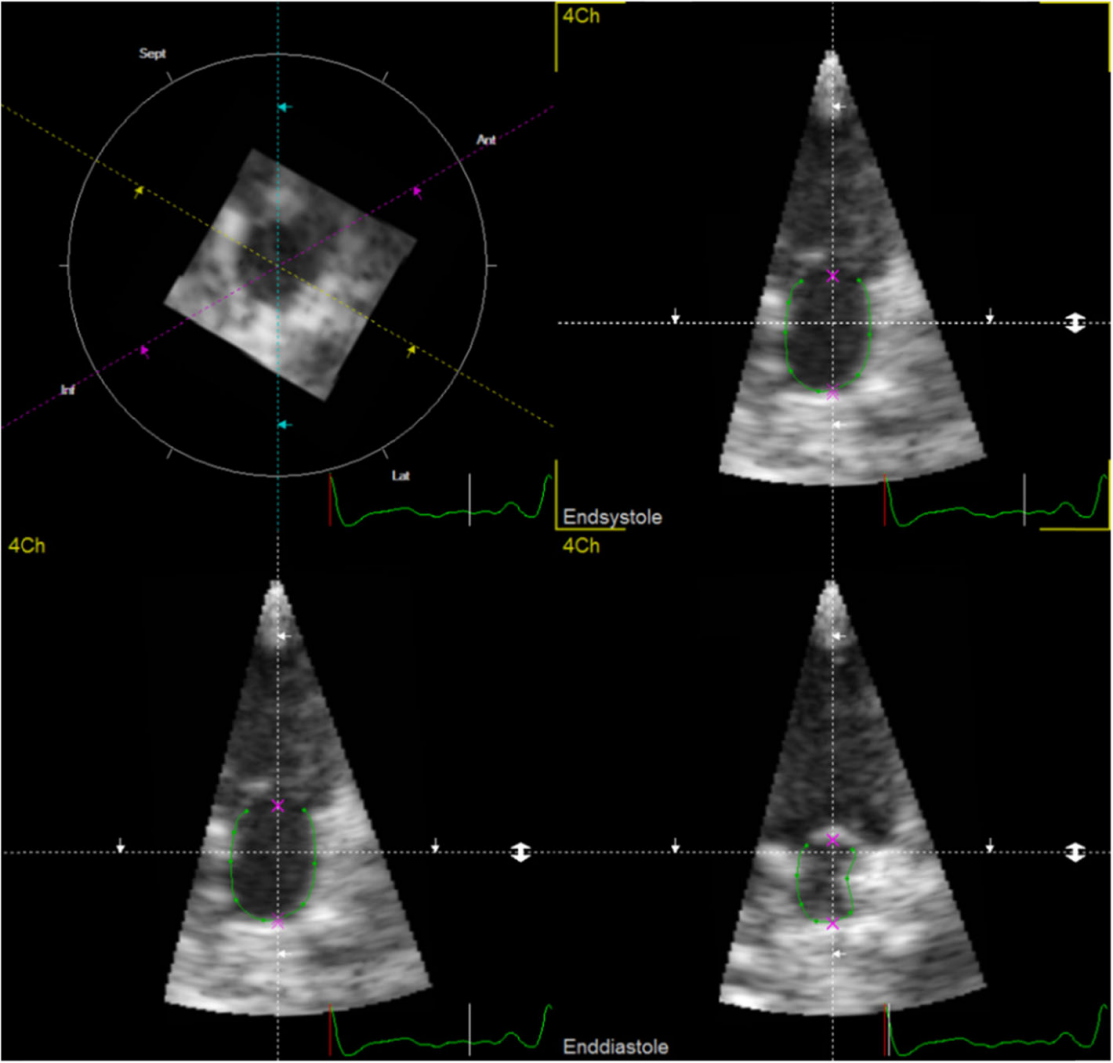
Left atrium (LA) volume measurement using Real Time 3 dimensional Echocardiography (RT3DE) with 4DE-TomTec™ as evaluation program: Representative images of LA volumetry in a healthy cat with RT3DE. The atrium is concurrently displayed in dynamic short axis view, left apical 4 chamber view, left apical 2 chamber view and left apical 3 chamber view

**Fig. 4:**
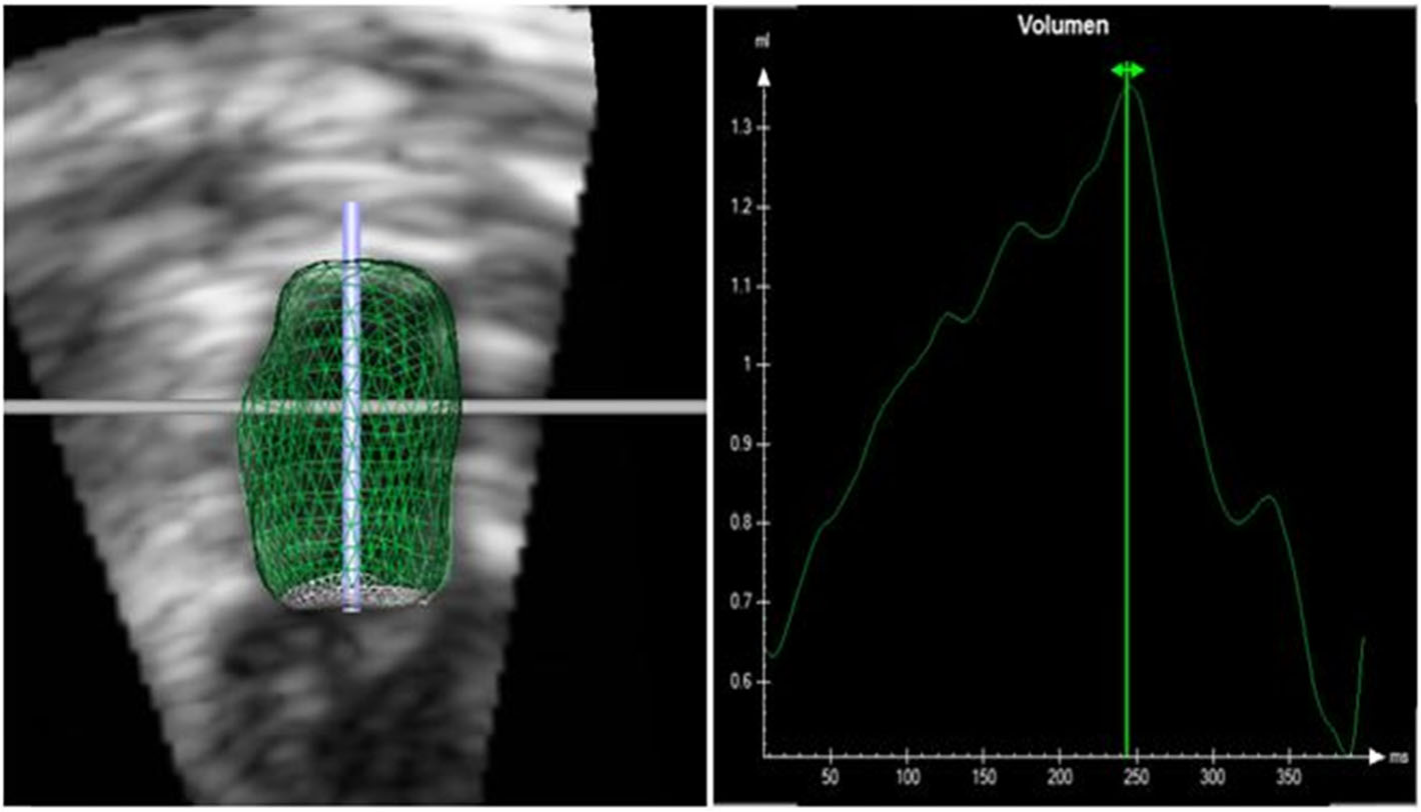
Left atrium (LA) volume measurement using Real Time 3 dimensional Echocardiography (RT3DE) and 4DE-TomTec™ as evaluation program: Representative RT3DE analysis of the left atrium in a healthy cat. The dynamic 3D model of LA as well as the time volume curve is displayed

RT3DE was performed in the left apical axis as described for RTTPE examinations [12]. The Vivid E9 is able to create a volume block by using multiple subvolumes of ECG triggered consecutive R-R cycles, to attain higher frame rates and therefore better spatial resolution. However, because of the small size of the feline heart, frame rates above 40 fps were reached by using just one sub volume. Therefore, we truly achieved a real time 3D examination. LAV quantifications were offline performed with the 4D-TomTec™ program.^3^ The first step was to centre LA manually in all planes to avoid foreshortening. The program was designed to quantify ventricles, so end-diastole and end-systole of the ventricle were detected automatically based on the ECG. In contrast to the RTTPE method, 4D-TomTec™ displayed one axis at a time with both, end-systole and end-diastole. The atrial endocardial border was detected automatically and had to be corrected manually for optimal tracing. It was made sure to exclude pulmonary veins as well as the left appendage. After tracing the endocardial border in these six pictures, a border detection of the whole heartbeat started. Manual adaption of the border lines in all frames of this heart beat were performed, in order to achieve the best alignment (Fig. 3). Finally, LA was reconstructed as a dynamic 3D body and a volume curve for the edited heart cycle was calculated (Fig. 4). Based on this volume curve, maximum and minimum volume were determined and SV and EF calculated.

### Time for measurements

The duration of each method was recorded with a stopwatch from the point of opening the data set for analysis until LAMin, LAMax, EF and SV were calculated.

### Statistical analysis

**Fig. 5:**
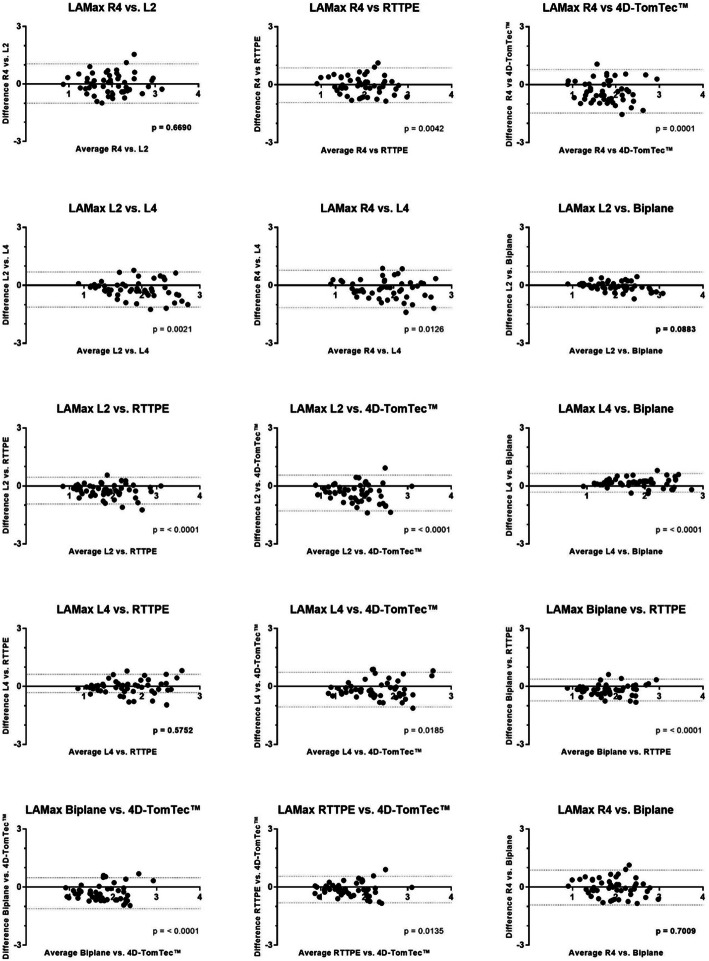
Bland-Altman plots comparing 6 different methods for left atrial maximum volume (LAMax) quantification: The methods are: monoplane Simpson method of discs (R4), left apical two chamber view (L2) and four chamber view (L4) measured with 2D monoplane Simpson method of discs, L2 and L4 combined to biplane volumetry (Biplane), Real Time Triplane Echocardiography (RTTPE), 4D-TomTec™ analysing software. Mean differences between the measurement-techniques were evaluated with a t-Test. P > 0.05 was evaluated as significant similarity and marked as bold letters

Statistics and graphs were calculated with commercially available software^4^^,.^^5^ All measurements were performed three times and averaged for statistical analysis. Normal distribution of LAMax, LAMin, EF and SV was verified with the Shapiro-Wilk method. The averaged values were expressed as mean ± standard deviation (SD) and as 95% prediction interval in ml. Bland-Altman graphs were created to visualize the relations between different measurement methods for LAMax (Fig. 5). Bias and Limits of Agreement (LOA) are summarized in Table 8. Since r4, l2 and biplane SMOD did not result in significantly different LAMax values, one reference range for the three methods was set up. All LAMax measurement data obtained by these three methods was used to calculate a common Mean and SD. A descriptive statistical analysis was used for age, body weight and sex. The influence of age and weight on the left atrial volume was calculated with the coefficient of determination R^2^. R^2^ = 1 is regarded as proof for perfect linear correlation, whereas R^2^ = 0 stands for no linear connection. To detect whether there are LA volume differences in male and female cats, simple T-test was calculated. The difference was considered significant when *p* < 0.05. For group-comparison single factor variance analyses as well as a paired T-test for multiple pairwise comparisons of the normal distributed data were performed. A *p*-value of less than 0.05 was considered significant. The duration of measurement for each method was normally distributed and therefore specified with mean ± SD, Min and Max. The increase of volume from female to male left atrial volume (LAV) was calculated with the following formula: $LAV\  Difference\ \left(\%\right)=\left(\frac{Mean\ Difference}{LAV\  Female}\right)\ast 100$. Inter- and intraobserver variability was evaluated by coefficient of variation (CV) for all methods. Intraobserver CV was calculated as mean divided by standard deviation and expressed as percentage. For interobserver variability, five patients were independently measured by two different observers. CV was then calculated as standard deviation of the mean difference between the two data sets divided by the total mean and multiplied by 100.

**Table 8:** Overview of Bland-Altman analysis results

Methods compared	Bias	upper LOA	lower LOA
R4 vs L2	0.0320	1.063	−0.9989
R4 vs L4	−0.1816	0.7899	−1.153
R4 vs Biplane	−0.025	0.8717	−0.9217
R4 vs RTTPE	−0.025	0.8717	− 0.9217
R4 vs 4DTomTec™	−0.3388	0.7891	−1.467
L2 vs L4	−0.2136	0.6981	−1.125
L2 vs Biplane	−0.057	0.3977	−0.5117
L2 vs RTTPE	−0.2442	0.4469	−0.9353
L2 vs 4DTomTec™	−0.3708	0.551	−1.293
L4 vs Biplane	0.1566	0.6319	−0.3187
L4 vs RTTPE	−0.0306	0.7212	−0.7824
L4 vs 4DTomTec™	−0.1572	0.7369	−1.051
Biplane vs RTTPE	−0.1872	0.3797	−0.7541
Biplane vs 4DTomTec™	−0.3138	0.4862	−1.114
RTTPE vs 4DTomTec™	−0.1266	0.5576	−0.8108

Results of Bland-Altman comparison between LAMax results of 50 healthy cats measured with 6 echocardiographic methods: right parasternal 4 chamber view measured with 2D monoplane Simpson method of discs (R4), left apical two chamber view (L2) and four chamber view (L4) measured with 2D monoplane Simpson method of discs, L2 and L4 combined to biplane volumetry (Biplane), Real Time Triplane Echocardiography (RTTPE) and 4D-TomTec™ analysing software. (LOA = limit of agreement)

## Data Availability

The datasets used and analysed during the current study are available from the corresponding author on reasonable request.

## Abbreviations

1DE: One dimensional echocardiography
2DE: Two dimensional echocardiography
3DE: Three dimensional echocardiography
CMRI: Cardiac MRI
ECG: Electrocardiogram
EDV: End-diastolic volume
EF: Ejection fraction
ESV: End-systolic volume
L2: Left apical two chamber view
L4: Left apical four chamber view
LA: Left atrium
LAA: Left atrial appendage
LAV: Left atrial volume
LA/AO: Proportion of the left atrial diameter to the aortic valve
LAAV: Left atrial appendage volume
LAMax: Left atrial maximum volume
LAMin: Left atrial minimum volume
LV: Left ventricle
MHZ: Megahertz
Ml: Mililiter
MM: Milimeter
MMode: Motion Mode
R4: Right parasternal four chamber view
R4AL: Right parasternal four chamber view calculated with Area/Length formula
RT3DE: Real Time 3D Echocardiography
RTTPE: Real-time triplane echocardiography
SD: Standard deviation
SMOD: Simpson method of discs

## Footnotes

1 Vivid E9, GE Healthcare, Horten, Norway.

2 EchoPAC PC, 108.1.4 Version 110.x.x, GE Healthcare, Horten Norway.

3 4D LV-Function™ 2.2, TomTec Imaging Systems GmbH, Unterschleißheim.

4 SAS version 9.3, SAS Institute, Cary, NC, USA.

5 GraphPad Prism, Version 5.00 forWindows, GraphPad Software, San Diego, Calif., USA. 4D-AutoLVQ™, EchoPAC PC, GE Healthcare, Horten, Norway.
